# Distal airway stem cells ameliorate bleomycin-induced pulmonary fibrosis in mice

**DOI:** 10.1186/s13287-019-1257-2

**Published:** 2019-06-03

**Authors:** Yun Shi, Mingqing Dong, Yueqing Zhou, Wangping Li, Yongheng Gao, Luyao Han, Min Chen, Hongwei Lin, Wei Zuo, Faguang Jin

**Affiliations:** 1 Department of Respiratory and Critical Care Medicine, Tangdu Hospital, Fourth Military Medical University, Xi’an, 710038 People’s Republic of China; 2 Xi’an International University, Xi’an, 710077 People’s Republic of China; 3 Shanghai East Hospital, School of Medicine, Tongji University, Shanghai, 200120 People’s Republic of China; 4 Kiangnan Stem Cell Institute, Zhejiang, 311300 People’s Republic of China; 5 Ningxia Medical University, Yinchuan, 750004 People’s Republic of China

**Keywords:** Pulmonary fibrosis, Distal airway stem/progenitor cells, Bleomycin

## Abstract

**Background:**

Idiopathic pulmonary fibrosis is characterized by loss of lung epithelial cells and inexorable progression of fibrosis with no effective and approved treatments. The distal airway stem/progenitor cells (DASCs) have been shown to have potent regenerative capacity after lung injury. In this work, we aimed to define the role of mouse DASCs (mDASCs) in response to bleomycin-induced lung fibrosis in mice.

**Methods:**

The mDASCs were isolated, expanded in vitro, and labeled with GFP by lentiviral infection. The labeled mDASCs were intratracheally instilled into bleomycin-induced pulmonary fibrosis mice on day 7. Pathological change, collagen content, α-SMA expression, lung function, and mortality rate were assessed at 7, 14, and 21 days after bleomycin administration. Tissue section and direct fluorescence staining was used to show the distribution and differentiation of mDASCs in lung.

**Results:**

The transplanted mDASCs could incorporate, proliferate, and differentiate into type I pneumocytes in bleomycin-injured lung. They also inhibited fibrogenesis by attenuating the deposition of collagen and expression of α-SMA. In addition, mDASCs improved pulmonary function and reduce mortality in bleomycin-induced pulmonary fibrosis mice.

**Conclusions:**

The data strongly suggest that mDASCs could ameliorate bleomycin-induced pulmonary fibrosis by promotion of lung regeneration and inhibition of lung fibrogenesis.

**Electronic supplementary material:**

The online version of this article (10.1186/s13287-019-1257-2) contains supplementary material, which is available to authorized users.

## Background

Idiopathic pulmonary fibrosis (IPF) is an idiopathic interstitial lung disease characterized by loss of lung epithelial cells and inexorable progression of fibrosis, which results in loss of normal lung architecture, respiratory failure, and eventual fatal outcome [1–3]. Currently, there is no effective therapy for pulmonary fibrosis for lack of known etiology and adequate knowledge of their pathogenic mechanisms [4]. Therefore, there is an urgent need for developing new methods for the treatment of IPF [5].

Recently, interest has accumulated regarding tissue regeneration as a new therapeutic strategy for lung fibrosis. Broadly, tissue regeneration is achieved by proliferation of common differentiated cells and/or by deployment of specialized stem/progenitor cells, which has been applied in some studies [6–10]. After lung damage, adult lung tissue has a remarkable capacity for regeneration, owing to a few facultative stem/progenitor cell populations that become activated in response to tissue damage [11, 12]. The distal airway stem/progenitor cells (DASCs) expressing basal cell-restricted transcription factor p63 and keratin-5 (KRT5) have been shown to have potent regenerative capacity after lung injury [13–16]. Influenza-induced lung injury activated DASCs to proliferate and migrate widely to occupy heavily injured areas, whereupon they differentiate toward mature epithelium and the regeneration of injured tissue depending on the extent of injury [14]. The feasibility for large-scale in vitro expansion and remarkable lung engraftment after transplantation make DASC an ideal candidate for cell therapy [15, 17].

In the current study, we aim to determine whether mouse DASC (mDASC) transplant has protective effects against bleomycin-induced lung fibrosis in mice. The results showed that mDASCs could incorporate, proliferate, and differentiate into type I pneumocytes. In addition, mDASCs could inhibit pulmonary fibrogenesis by attenuating the deposition of collagen and the expression of α-SMA in lungs. Furthermore, mDASCs improved the pulmonary function and reduced the animal’s mortality in bleomycin-induced pulmonary fibrosis mice. The results indicated that DASCs may be an ideal candidate for the cell therapy of pulmonary fibrosis.

## Methods

### Isolation and culture of mDASCs

The isolation and culture of mDASCs was based on previously described methods [15]. Briefly, the lung tissue of adult mice was collected and immersed in cold wash buffer (F12 medium, 1% Pen/Strep, 5% FBS). The trachea and two main bronchi were separated from the lungs, and the lobes were cut with a sterile surgical blade into small pieces and digested with dissociation buffer (F12/DMEM, 1 mg/mL protease, 0.005% trypsin and 10 ng/mL DNaseI) overnight with gentle rocking. Dissociated cells were passed through 70-μm Nylon mesh, washed with cold F12 medium, and then plated onto irradiated 3T3 feeder cells as described. Under 7.5% CO_2_ culture condition, the mDASC colonies emerged 3–5 days after plating. Cells were digested by 0.25% trypsin-EDTA (Gibco, USA) for 3–5 min for passaging. To label cells with GFP, pLenti-CMV-EGFP plasmid was transfected into 293T cells together with lentivral packaging mix (Life Technologies, USA). Lentivirus supernatant produced by 293T was collected, filtered, and cryo-preserved before use. To infect mDASCs, 0.5 mL lentivirus containing medium was directly added to 2 mL cell culture medium with 10 μg/mL polybrene and incubated for 12 h, and the overall labeling efficiency of cells was above 95%.

### Differentiation of mDASCs in vitro

The mDASCs were seeded on Matrigel Matrix (Corning, USA) as previously described [13]. Cells were cultured in serum-free DMEM/F12 medium for 7 days, adding FGF10 (50 ng/mL, Peprotech, USA), transferrin (5 μg/mL, Peprotech, USA), HGF (20 ng/mL, Peprotech, USA), 2% Matrigel, and 5% BSA. Differentiated organoids were harvested and embedded in Tissue-Tek O.C.T. Compound (Sakura, USA), then the differentiation characteristics of cells were identified by immunofluorescence.

### Mice, bleomycin injury models, and treatment protocols

Six- to 8-week-old female C57/B6 mice were purchased from Vital River (China) and were housed in the SPF animal facility. All studies were approved by the Institutional Animal Care and Use Committee of the Fourth Military Medical University. Mice were anesthetized with isoflurane, the lung was injured by intratracheally instilling with 3 U/kg bleomycin (Selleckchem, USA) in the volume of 30 μl on day 0, and the weight of the mice was recorded 7, 14, and 21 days after injury. One million GFP-labeled mDASCs in a volume of 30 μl or saline were transplanted on day 7 after injury. Intratracheal aspiration was performed by injecting the cells into the trachea via the mouth which was described in our previous publications [15]. The mDASCs were counted, washed, and resuspended by PBS before transplantation. Bright-field and direct fluorescence of the transplanted lung were acquired under the fluorescence stereomicroscope (MVX10, Olympus, Japan).

### Lung wet-to-dry weight (W/D) ratios

To quantify the magnitude of pulmonary edema caused by bleomycin, we evaluated the wet-to-dry ratios. The lungs were excised at an indicated day after injury, and the wet weight was recorded. The dry weights were obtained after the lungs were dried in an oven at 70 °C for 72 h. The W/D ratios were then calculated.

### Tissue histology

At appropriate time points, mice were euthanized and the diaphragm was carefully cut open without touching the lung. The lung was inflated with 3.7% formaldehyde (Sigma, USA) using a 30-G needle through the trachea. Then the lung was dissected and fixed in 3.7% formaldehyde at 4 °C overnight before paraffin section or cryosection. For cryosection, the fixed lung was settled by 30% sucrose before embedding into the Tissue-Tek O.C.T compound (Sakura, Japan), solidified on dry ice, and cut using a cryotome (Leica Microsystems, Germany) of 5–10-μm thickness. For the paraffin section, the lung was dehydrated by gradient ethanol in an automatic tissue processer (Leica Microsystems, Germany) and then embedded into paraffin blocks. The blocks were cut into 5~7-μm thickness by using a microtome (Leica Microsystems, Germany) at distinct planes. The sections were placed on poly-lysine-coated glass slides and stored at room temperature until further use. Hematoxylin and eosin (H&E) and Masson’s trichrome staining was performed following the standard protocol. The severity of fibrosis in H&E-stained lungs was quantified by the Ashcroft scoring system [18], and the blue-stained area (an indication of quantity of collagen deposition) was separately quantified by ImageJ version 1.52a (National Institutes of Health, USA).

### Immunofluorescence staining

For immunofluorescence staining, cryo-embedded tissue slides were subjected to antigen retrieval in citrate buffer (pH 6, Sigma-Aldrich, USA) at 120 °C for 20 min, and 10% normal donkey serum (Jackson Immuno Research) was used to block the non-specific antigen. Antibodies used for immunofluorescence included mDASC markers: KRT5 (1:200, ab128190, Abcam) and P63 (deltaN,1:200, 4A4, Abcam); pneumocyte markers: PDPN (1:200, FL-162, Santa Cruz), AQP5 (1:200, EPR3747, Abcam), and HOPX (1:200, E-1, Santa Cruz); myofibroblast marker: α-SMA (1:500, 1A4, DAKO); and others: GFP (1:1000, ab5450, Abcam) and KI67 (1:200, RM-9106, Thermo). Alexa Fluor-conjugated Donkey 488/594/647 (1:200, Life Technologies, USA) were used as secondary antibodies. After counter staining with DAPI (Roche, USA), samples were treated with 0.1% Sudan Black (Sigma, USA) for 1–2 min to remove autofluorescence and then mounted with VECTASHIELD® Mounting medium (Vector Labs, USA). Stained slides were stored at 4 °C in the dark, and images were taken by using a fluorescence microscope (Nikon 80i and Eclipse Ti, Nikon, Japan).

### Measurement of lung hydroxyproline

To evaluate the extent of tissue fibrosis, we measured the total collagen contents using a hydroxyproline assay kit (Nanjing Jiancheng Bioengineering Institute, China). The experimental procedure was according to the manufacturer’s instructions.

### Western blotting

The tissues were collected and homogenized immediately, and the protein was extracted using the Total Protein Extraction Kit. The BCA protein assay kit was used to determine the protein concentration. Twenty micrograms total protein was loaded and separated on a 10% SDS poly-acrylamide gel, and then transferred to the PVDF membrane (Roche). The membrane was blocked with 5% non-fat dry milk for 2 h, followed by incubation with primary antibodies against α-SMA (1:500, 1A4, DAKO) or GAPDH (1:500, GB12002, Servicebio) overnight. Then the membrane was incubated with the secondary antibody. The specific signals were detected by the Immobilon Western Chemiluminescent HRP Substrate (Millipore) and Tanon image system.

### Arterial blood gas measurements

Mice were anesthetized at appropriate time points, and the blood samples were drawn from the carotid aorta into polypropylene syringes containing 60 IU of dry, electrolyte-balanced heparin (PICO70; Radiometer Medical, Copenhagen, Denmark). The partial oxygen pressure (pO_2_), partial carbon dioxide pressure (pCO_2_), and oxygen saturation (sO_2_) were measured using an ABL90 Flex Blood Gas Analyzer (Radiometer Medical). Independent DASC isolations were used, and the engraftment levels of GFP-mDASC were evaluated under a stereo fluorescence microscope. Only the animals with similar levels of engraftment of GFP-mDASC were used for analysis.

### Survival studies

For the assessment of mortality rates, mice were given 5 U/kg bleomycin by intratracheal aspiration on day 0 and transplanted by mDASCs or saline on day 7. The mortality of mice in each group was recorded every day for 24 days after bleomycin administration.

### Statistics

Statistical calculations were performed using GraphPad Prism (GraphPad Software, Inc., San Diego, CA, USA). Comparisons between two groups were made by an unpaired *t* test. Comparisons between more than two groups were analyzed by one-way ANOVA or two-way ANOVA with post Tukey’s multiple comparisons test. Survival data were presented by the Kaplan–Meier method, and comparisons were made by the log rank test. All values were expressed as means ± SEM. *P < 0.05 and **P < 0.01 were considered statistically significant.

## Results

### Characterization of endogenous mDASCs in bleomycin-induced lung injury in mice

**Fig. 1:**
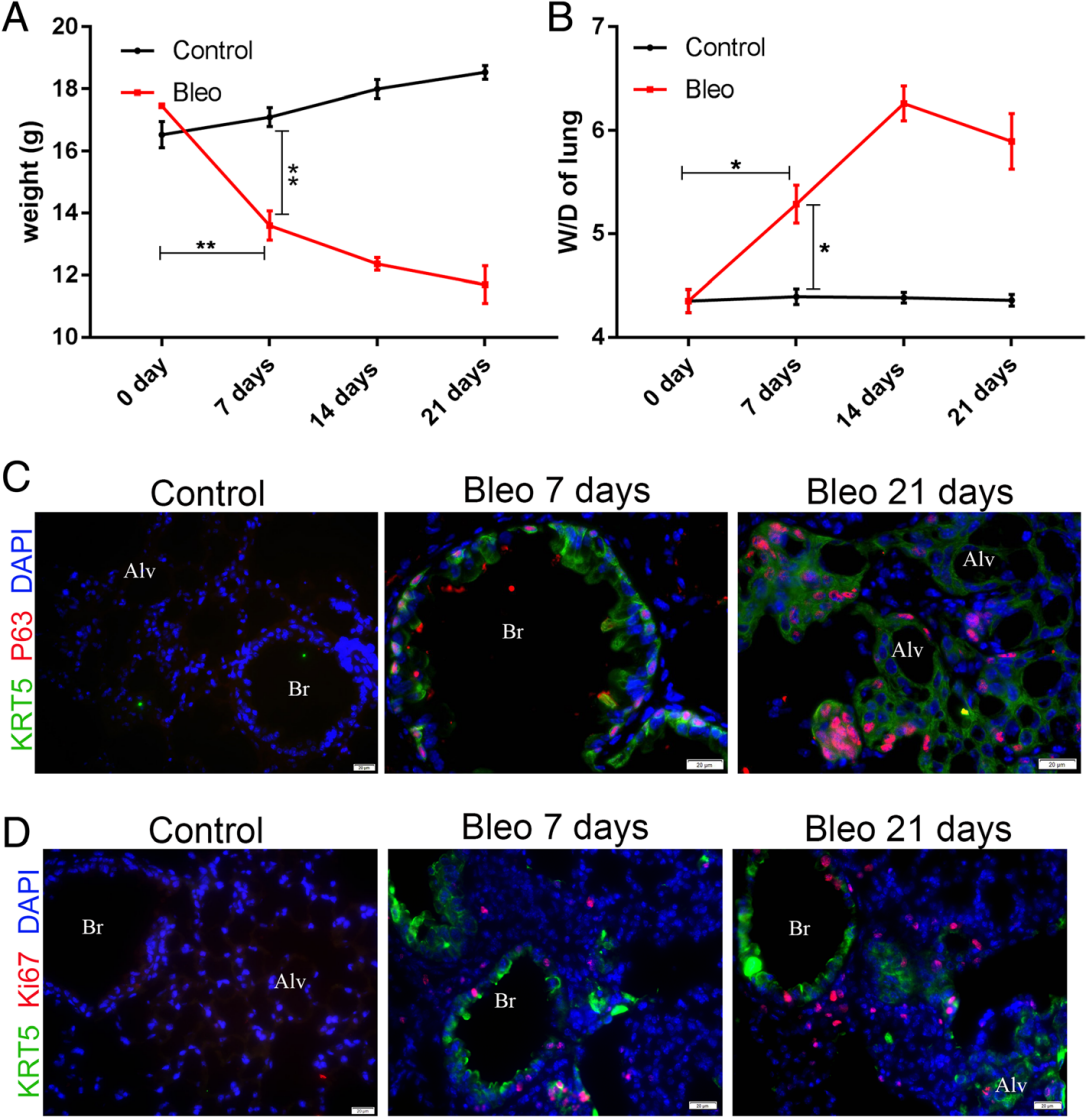
Characterization of endogenous mDASCs in bleomycin-induced lung injury in mice. **a** The weight changes of mice after the delivery of bleomycin. *n* = 3. Error bars, S.E.M. **P < 0.01. **b** The lung wet-to-dry weight ratios induced by bleomycin. *n* = 3. Error bars, S.E.M. *P < 0.05. **c** Anti-KRT5 and anti-P63 immunofluorescence staining of a lung section 7 and 21 days after administration of bleomycin. **d** Anti-KRT5 and anti-KI67 immunofluorescence staining of a lung section 7 and 21 days after administration of bleomycin. Br, bronchioles; Alv, alveoli. Scale bar, 20 μm

Delivery of bleomycin to the lung caused acute pulmonary injury that was mirrored by the changes of weight and wet/dry ratios. The weight of pulmonary injury model mouse decreased, but the weight of control mouse increased significantly during the experiment (Fig. 1a). The weight was significantly different between the control mouse and model mouse at 7, 14, and 21 days after bleomycin delivery (all *P* < 0.05, *n* = 3). The lung wet/dry ratios of the injury mouse significantly increased 7, 14, and 21 days after bleomycin administration compared with those of the control mouse (all *P* < 0.05, *n* = 3) (Fig. 1b).

It had been demonstrated that the expression of KRT5 and P63 was the characteristic marker of distal airway stem cells; we found little evidence of KRT5+P63+ cells in the parenchyma of normal mice. However, 7 days after administration of bleomycin, some KRT5+P63+ cells could be found in the bronchioles by immunofluorescence; these cells in the damage lung appeared to cluster in small groups and are distributed in a concentric pattern about the bronchioles, and these cells also could be found in broader regions of the alveolar space as time went on (Fig. 1c, Additional file 1: Figure S1a and b). A fraction of these cells expressed proliferation marker Ki67 (Fig. 1d and Additional file 1: Figure S1c), suggesting their high viability and extended contribution to the repair process.

### Transplanted mDASCs incorporated in damaged lung and differentiated into type I pneumocytes

Although the endogenous KRT5+P63+ cells could be found in broader regions of the alveolar space as time went on, their number was still less and not enough to inhibit the progression of bleomycin-induced fibrosis (Additional file 2: Figure S2a). Therefore, we investigated the effect of exogenous mDASCs by transplanting them into the lungs of bleomycin-injured mice.

**Fig. 2:**
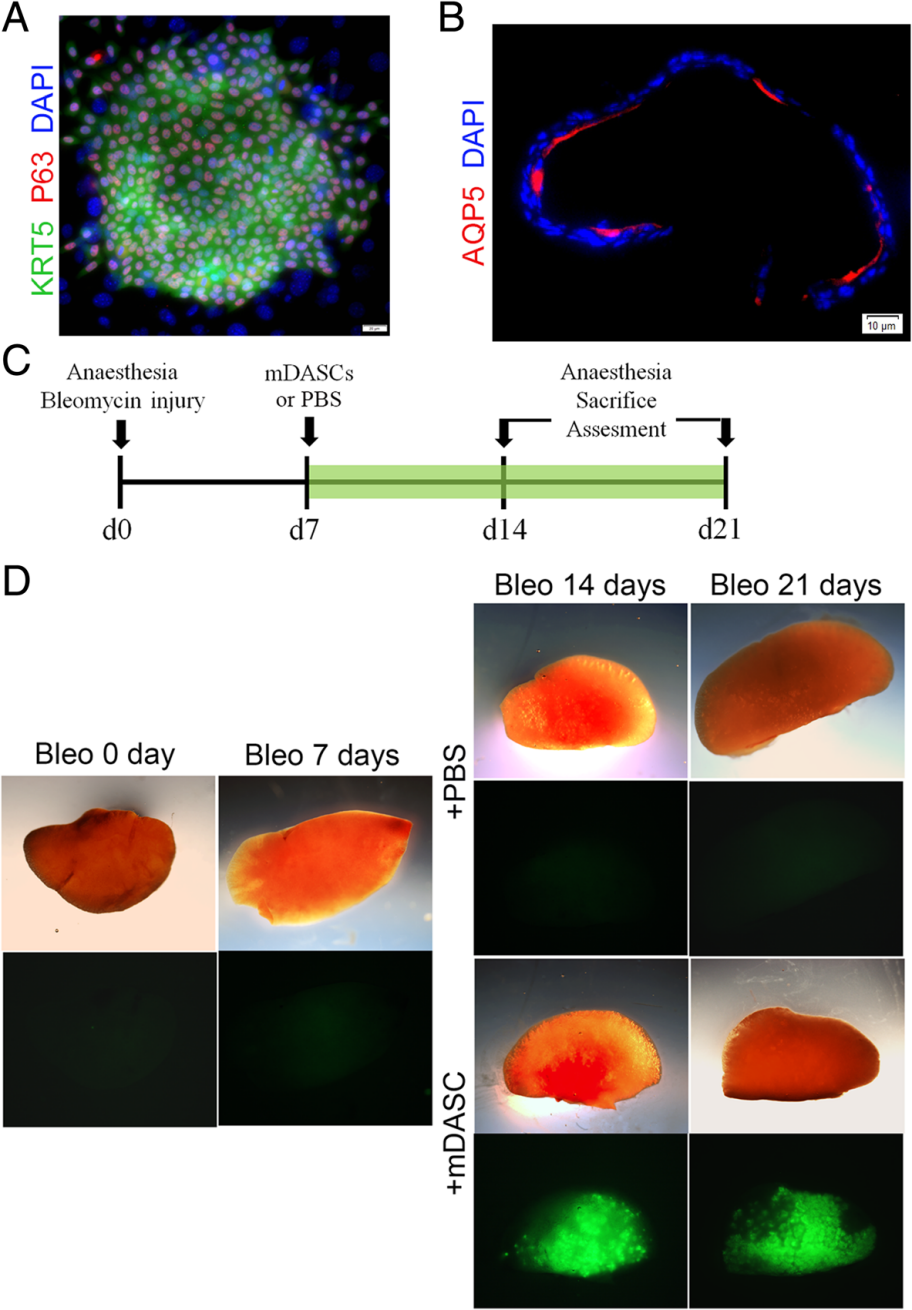
The mDASCs differentiated into type I pneumocytes in vitro and incorporated in damaged lung. **a** Anti-KRT5 and anti-P63 immunostaining of mDASC colonies with nuclei counterstain. Scale bar, 20 μm. **b** Differentiation of mDASCs in three-dimensional Matrigel cultures showing an alveolar-like sphere structure and expression of AQP5 (type I alveolar cell marker). Scale bar, 10 μm. **c** Schematic diagram of the experiment. **d** Bright-field and direct fluorescence image of mouse lungs following transplantation of 1 × 10^6^ GFP-labeled mDASCs on the indicated days after bleomycin injury

To make sure that the transplanted cells were DASCs but not just other basal cells, which also express P63 and Krt5, the transplanted mDASCs were identified by culturing in Matrigel. The mDASCs expressed the markers of P63 and KRT5 (Fig. 2a), which formed an alveolar-like sphere structure with the type I alveolar cell marker AQP5 expression during a 7-day period of culture (Fig. 2b), which was consistent with previous studies [15].

**Fig. 3:**
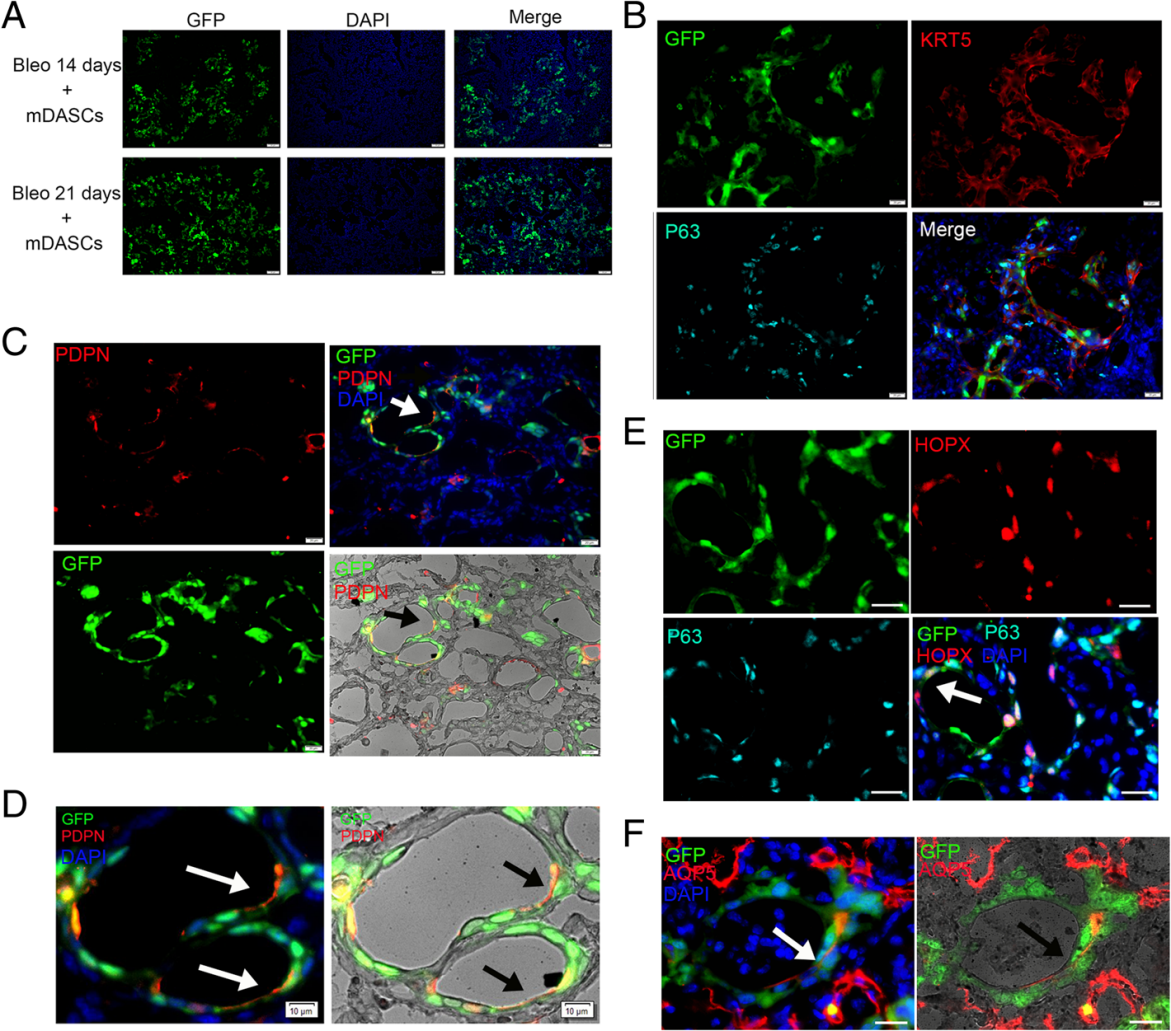
Transplanted mDASCs incorporated in damaged lung and differentiated into type I pneumocytes. **a** Distribution of transplanted GFP-labeled cells in lung parenchyma by direct fluorescence. Scale bar, 50 μm. **b** Immunostaining of GFP-labeled mDASCs with anti-GFP, anti-KRT5, and anti-P63 antibodies 7 days after transplantation. Scale bar, 20 μm. **c** Immunostaining of transplanted GFP-labeled mDASCs in the lung parenchyma with anti-GFP and anti-PDPN (type I alveolar cell marker) antibodies 14 days after transplantation. Scale bar, 20 μm. **d** Amplification inset in **c** indicates a regenerated alveolar structure. Scale bar, 10 μm. **e** Immunostaining of transplanted GFP-labeled mDASCs in the lung parenchyma with anti-GFP, anti-HOPX (type I alveolar cell marker), and anti-P63 antibodies 14 days after transplantation. Scale bar, 20 μm. **f** Immunostaining of transplanted GFP-labeled mDASCs in the lung parenchyma with anti-GFP and anti-AQP5 (type I alveolar cell marker) antibodies 14 days after transplantation. Scale bar, 20 μm. Arrows show the co-localization staining of GFP and indicated marker

Then, we investigated the effect of mDASCs by isolating, culturing, and transplanting them into the lung of mice. One million GFP-labeled mDASCs were transplanted into the lung 7 days after bleomycin injury. The tissues were harvested for analysis on 7 and 14 days after cell transplantation (Fig. 2c). Substantial incorporation of mDASCs (and their descendants) into mouse lung was detected along the days (Fig. 2d). Direct fluorescence of tissue section showed distribution of GFP-mDASCs in mouse lung parenchyma, and a fraction of mDASCs differentiated into an alveolar-like structure in later days (Fig. 3a). Expectedly, we observed the incorporation of mDASCs with expression of KRT5 and P63 7 days after transplantation (Fig. 3b). A few cells also formed air sacs with the expression of type I pneumocyte markers PDPN (Fig. 3c, d), HOPX (Fig. 3e), and AQP5 (Fig. 3f) 14 days after transplantation. Taken together, these findings demonstrate that mDASCs could incorporate in the damaged lung and differentiated into type I pneumocyte cells which were contributory to the repair process.

Interestingly, we found no incorporation of mDASCs when they transplanted into the normal lung (Additional file 2: Figure S2b), the weight changes (Additional file 2: Figure S2c), and lung wet/dry ratios (Additional file 2: Figure S2d) of mice showed that mDASCs had no effect on normal lungs, which was confirmed by tissue histology (Additional file 2: Figure S2e). We also transplanted the basal cells isolated from the mouse cervix epithelium to the lung 7 days after bleomycin injury as a control to determine the paracrine effect of mDASCs. However, we did not find their incorporation in the injured lungs 7 days after transplantation (Additional file 2: Figure S2f). These data indicated the special incorporation ability of mDASCs in damaged lung.

### Transplanted mDASCs decreased the pulmonary fibrosis in a bleomycin mouse model

**Fig. 4:**
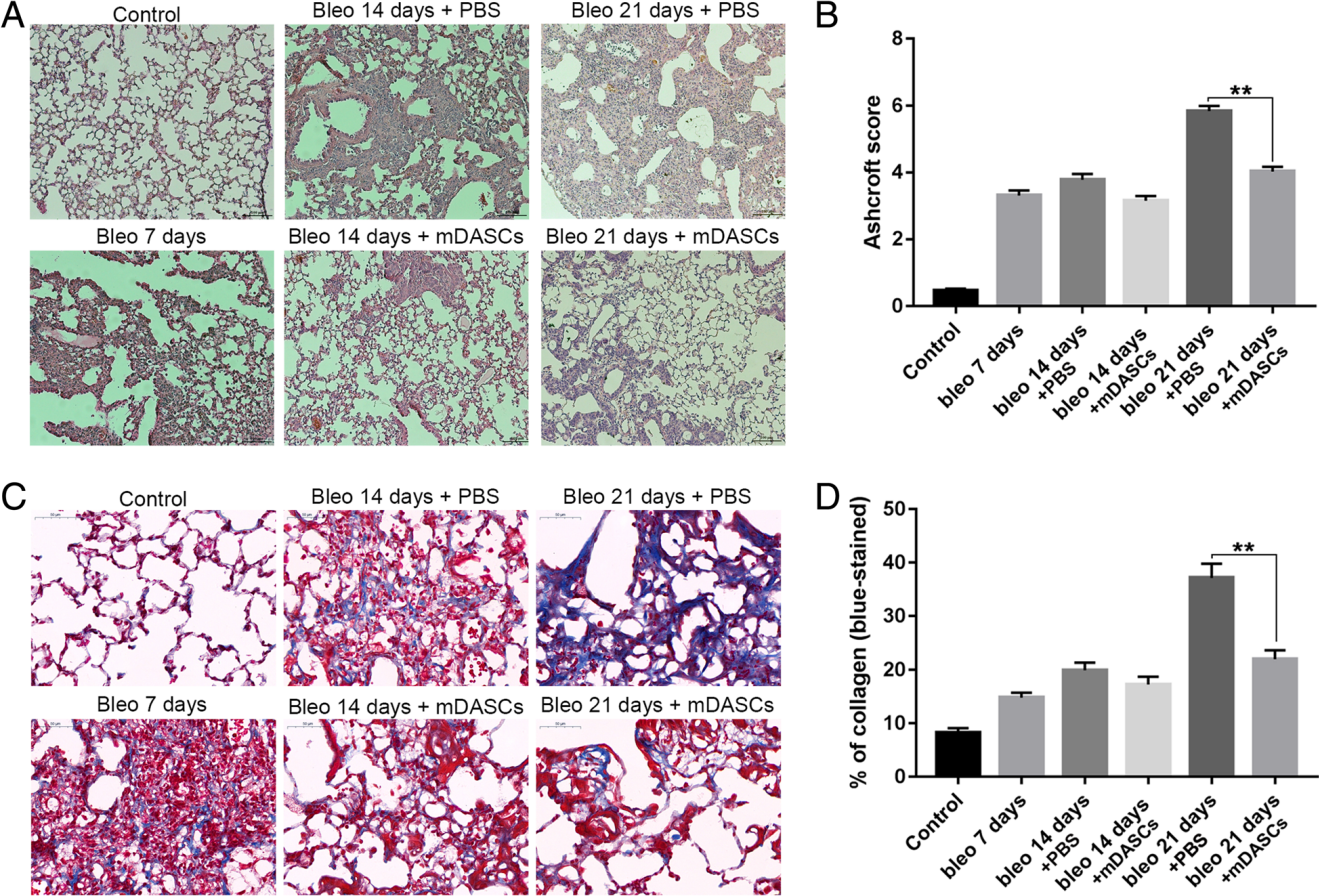
The protective effect of transplanted mDASCs on bleomycin-induced pulmonary fibrosis development. **a** The mDASCs were transplanted into recipient mice 7 days after bleomycin instillation, and the lung sections were stained by hematoxylin and eosin (H&E) 7, 14, and 21 days after bleomycin instillation. Scale bar, 100 μm. **b** Ashcroft score of lung fibrosis for panel **a**. *n* = 4. Error bars, S.E.M. **P < 0.01. **c** Using the same experimental setting, the lung sections were stained by Masson’s trichrome for collagen I deposition (blue). Scale bar, 50 μm. **d** Quantitation of the collagen content for panel **b**. *n* = 5. Error bars, S.E.M. **P < 0.01

To determine how mDASCs affect pulmonary fibrogenesis, the extent of pulmonary fibrosis was analyzed. Representative microphotographs of H&E staining are shown in Fig. 4a. In the control groups, lung tissues showed a normal structure and clear pulmonary alveoli. Seven days after bleomycin administration, the lung tissue displayed obvious inflammatory cell infiltration, vascular congestion, thickened septa, and alveolar collapse. Fibrosis is fully developed with extensive and diffuse involvement of the lung at 21 days. In contrast, pulmonary fibrosis tended to decrease and the lesions were significantly attenuated when administrated with mDASCs (Fig. 4b). Masson’s trichrome staining of lung tissue (Fig. 4c) showed that more prominent blue staining, which represents mature collagen, was distributed in the alveolar septa or interstitial and peribronchial connective tissue in the bleomycin-injured lung without mDASC transplantation than that in the lung with mDASC transplantation (Fig. 4d). These findings support that engraftment of mDASCs 7 days after bleomycin instillation protected pulmonary fibrosis on days 14 and 21.

**Fig. 5:**
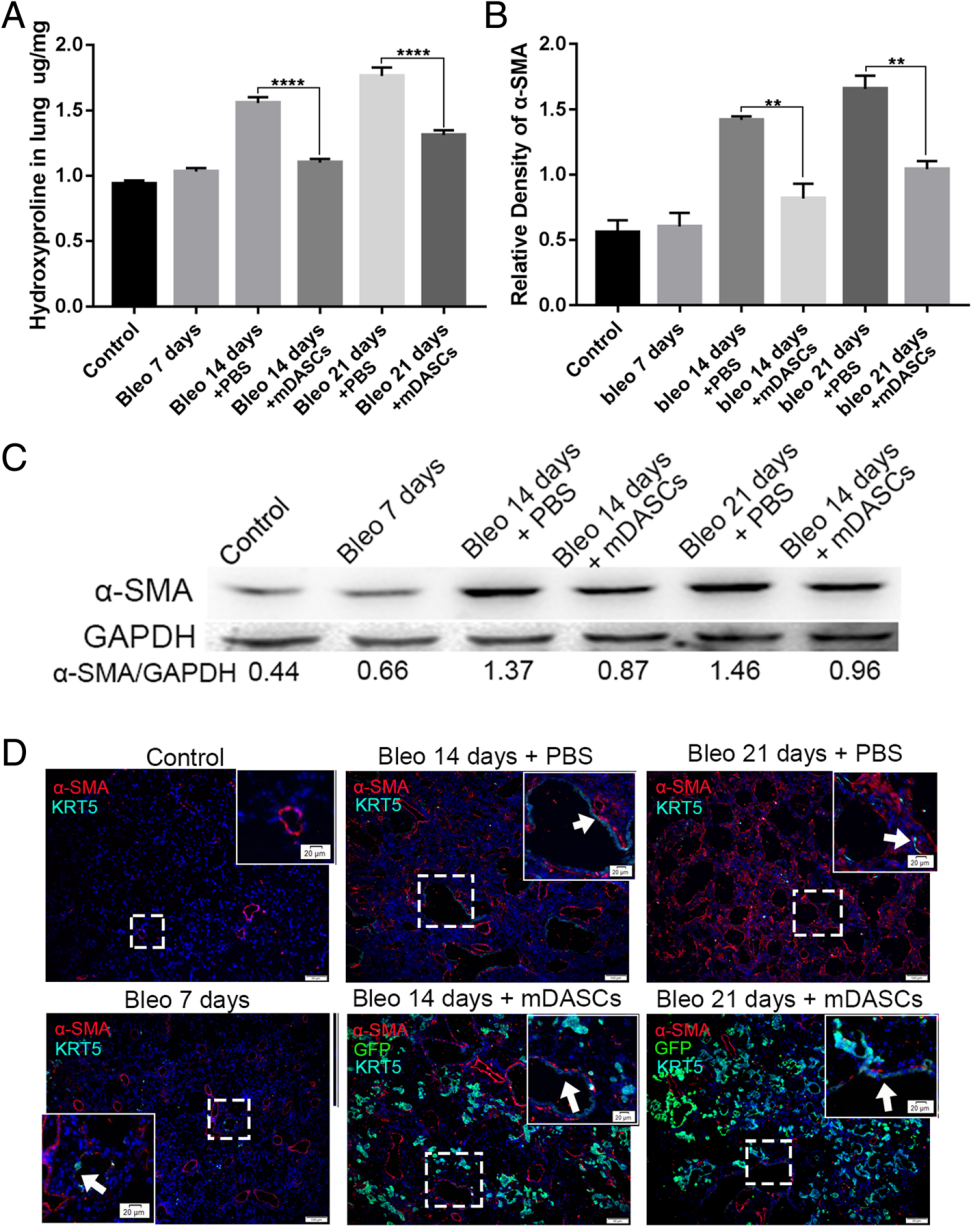
Transplanted mDASCs decreased the collagen content and expression of α-SMA in lungs in bleomycin-induced pulmonary fibrosis mice. **a** Collagen deposition was assessed by measuring the hydroxyproline content in lung tissue. *n* = 5. Error bars, S.E.M. **P < 0.01. **b**, **c** Western blotting of α-SMA expression and its quantification in lung tissues challenged by bleomycin with or without mDASC treatment. Samples were obtained at 7, 14, and 21 day after bleomycin exposure. *n* = 3. Error bars, S.E.M. **P < 0.01. **d** Immunofluorescence of α-SMA in a lung section 7, 14, and 21 days after bleomycin exposure treated with or without mDASCs. Scale bar, 50 μm. The endogenous mDASCs are indicated by arrows. Scale bar, 50 μm and 20 μm in amplification inset

We also examined the content of hydroxyproline, a major constituent of collagen in the lung tissues (Fig. 5a). Compared with the control group, hydroxyproline content of the lung significantly increased in the bleomycin-treated group. However, administration of mDASCs significantly inhibited the hydroxyproline accumulation induced by bleomycin. Accumulation of α-SMA is a hallmark of pathological remodeling in pulmonary fibrosis, so the expression of α-SMA protein was examined at different time points by Western blotting (Fig. 5b, c) and immunofluorescence (Fig. 5d). After bleomycin instillation, acute lung inflammation developed (day 7), and at this stage, we observed no change of α-SMA expression compared with the control group, but the expression of α-SMA increased following bleomycin treatment in lung tissues on days 14 and 21, which were significantly alleviated by treatment with mDASCs.

To determine if the observed effect of mDASCs was associated with secreted factors, we assessed the ability of the conditioned medium (CM) of mDASCs to inhibit hydroxyproline accumulation by intratracheally instilling with 30 μl CM 7 days after bleomycin injury, and the hydroxyproline content was measured 14 days after administration of CM. There was no significant effect of CM from mDASCs on hydroxyproline content in comparison with the control medium (with no cell) (Additional file 2: Figure S2 g).

### Transplanted mDASCs improved pulmonary function in bleomycin-induced pulmonary fibrosis mice

**Fig. 6:**
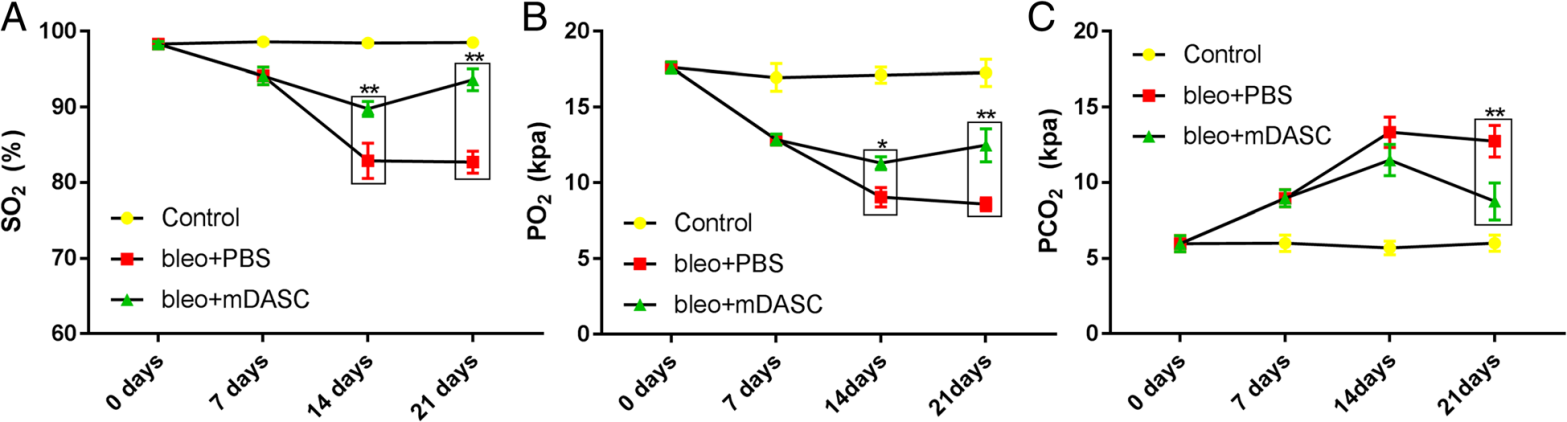
Transplanted mDASCs improved pulmonary function in bleomycin-induced pulmonary fibrosis mice. **a** Arterial blood gas analysis of O_2_ saturation (sO_2_) at appropriate time points while mice were challenged by bleomycin with or without mDASC transplantation. *n* = 4. Error bars, S.E.M. **P < 0.01. **b** Arterial blood gas analysis of O_2_ partial pressure (pO_2_). *n* = 4. Error bars, S.E.M. *P < 0.05, **P < 0.01. **c** Arterial blood gas analysis of CO_2_ partial pressure (pCO_2_). *n* = 4. Error bars, S.E.M. **P < 0.01

As presented in Fig. 6, bleomycin instillation markedly reduced O_2_ saturation and O_2_ partial pressure while it increased CO_2_ partial pressure in arterial blood in 7, 14, and 21 days. In contrast, the mice administrated with mDASCs demonstrated healthier post-injury pulmonary function as shown by the higher O_2_ saturation and O_2_ partial pressure, yet lower CO_2_ partial pressure. The data above showed that the mDASC transplantation could protect mouse lung and improve the pulmonary function of the recipients.

### The protective effect of transplanted mDASCs on bleomycin-induced mortality in mice

**Fig. 7:**
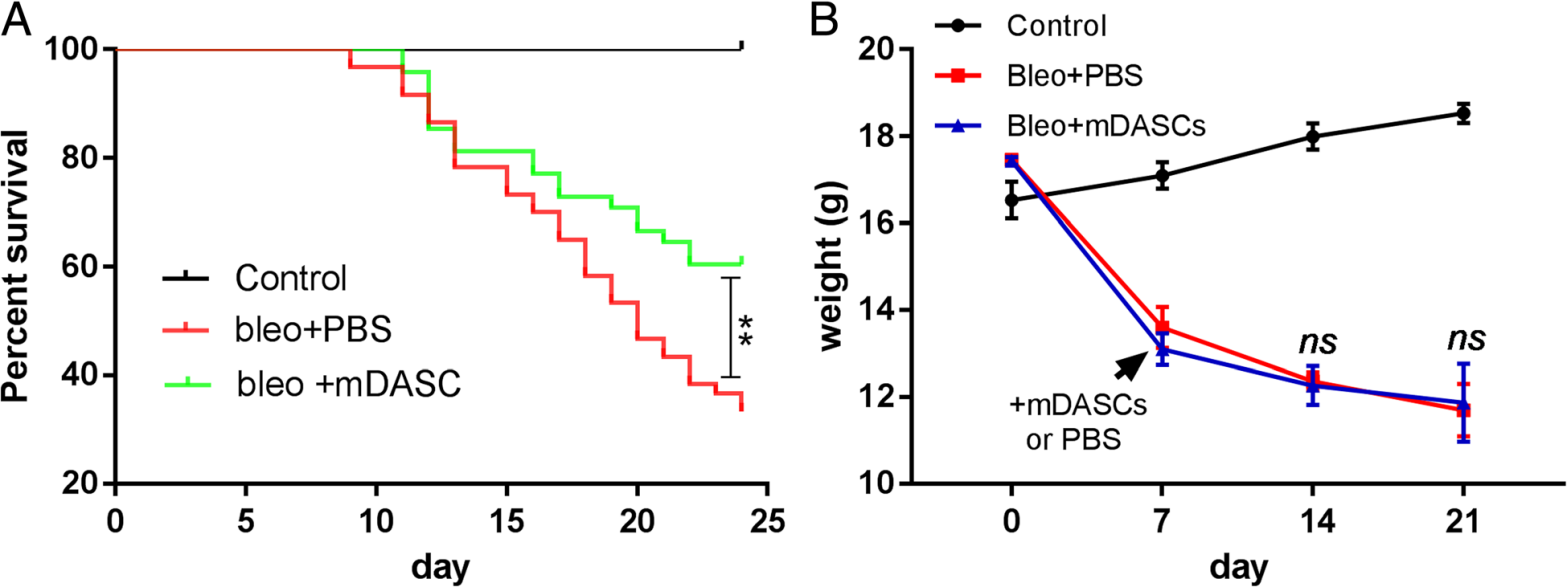
The protective effect of transplanted mDASCs on bleomycin-induced mortality in mice. **a** Mice were challenged by bleomycin (5 U/kg) with or without mDASC treatment, and mDASCs prevented the lethality induced by bleomycin. Survival was monitored every day during 24 days, and the percent survival rate was expressed as Kaplan–Meier survival curves. *n* = 10 (control), *n* = 60 (bleo), *n* = 48 (bleo + mDASCs). Error bars, S.E.M. **P < 0.01. **b** The weight changes of mice after the administration of mDASCs. *n* = 3. Error bars, S.E.M. *ns*, no significantly different

To evaluate the protective effect of mDASCs on bleomycin-injured mice, animals were given a lethal dose of bleomycin (5 U/kg) on day 0 and mDASCs (1 × 10^6^ in the volume of 30 μl) or the same volume of PBS was instilled into the lung on day 7. As shown in Fig. 7a, mice began to die 9 days after the administration of bleomycin; the accumulative mortalities during 24 days were 40% in mDASC treatment groups, which were significantly lower than those in PBS administration groups (67%). Mice administrated with mDASCs could survive over 40 days, although mDASCs had no significant effects on the body weight of bleomycin-injured mice (Fig. 7b). These data indicated that mDASCs could significantly reduce bleomycin-induced mortality in mice.

## Discussion

The present study firstly demonstrated that the transplantation of mDASCs at the acute injury stage could ameliorate the following fibrosis, improve lung function, and reduce mortality in bleomycin-induced fibrosis mice. Therefore, DASCs could be an ideal candidate stem cell for the therapy of pulmonary fibrosis.

Tissue-specific stem cells have been identified as multipotent cells with the capacity for long-term self-renewal and the ability to differentiate into other cell lineages. These stem cells are typically quiescent in normal conditions and proliferate during injury repair [19–21]. Endogenous adult lung stem cells are important for epithelial cell homeostasis and injury repair which have attracted significant interest. Numerous studies of human and animal lung have identified subsets of lung epithelial cells that could respond to lung injury with self-renewal and differentiation capacity, including basal cells, club cells, and AECII cells of the alveoli [12, 22, 23]. Moreover, advanced lineage tracing techniques have suggested that most types of lung epithelial cells can proliferate and expand after injury to promote lung repair [24–28]. Several studies demonstrated that P63+/KRT5+ mDASCs underwent rapid proliferation and migration to damaged alveolar regions in response to influenza-induced ARDS. These migrated cells assembled and expressed typical alveoli-associated markers which suggested that mDASCs played a role as an intermediate in the reconstitution of the alveolar-capillary network eradicated by viral infection [13–15].

A previous study reported that human KRT5+ basal cells were frequent in IPF distal lungs compared with healthy lungs and these basal cells expressed differentiated epithelial cell markers, indicating that they were likely attempting to regenerate the epithelium [29]. As expected, we observed the proliferating endogenous mDASCs^p63/Krt5^ in the damage lung in bleomycin-induced lung injury mice, which were hard to find in the healthy lung.

Different animal models have been developed to investigate key mechanisms underlying pathogenesis of pulmonary fibrosis and identify potential therapeutic targets. The most common pulmonary fibrosis model involves exposure to bleomycin, and several studies reported that administration of bleomycin to the lung causes pulmonary injury and inflammation, and then a chronic fibrotic process develops [30–32], which is characterized by replacement of extracellular matrix by fibrillary collagen and collagen-producing myofibroblasts [33]. Our findings showed that bleomycin induced a significant acute lung injury in 7 days and then caused obvious fibrosis in day 21. Therefore, these findings were consistent with the previous studies. The mDASCs were applied in the acute injury stage.

In our experiments, we observed large-scale incorporation of transplanted mDASCs into mouse lung. The engrafted mDASCs differentiated into alveolar-like structure in 21 days, and a fraction of them expressed type I pneumocyte markers but not the type II pneumocyte marker. However, Zuo et al. reported that mDASCs expressed type I and type II pneumocyte markers 40 days after virus infection [15], and Vaughan et al. also found that ~ 1/3 of the Krt5+ cells resolved into type II pneumocytes 50 days after bleomycin injury [14]. The difference of differentiation time of transplanted mDASCs in animals between our experiment and theirs maybe the main cause. Together, these findings demonstrated that the mDASCs could incorporate, proliferate, and differentiate into type I pneumocyte cells which indicated the participation of regeneration and reparation following lung injury.

Furthermore, our results showed that administration of mDASCs 7 days after bleomycin instillation ameliorated the histopathological characteristics of lung tissues and prevented pulmonary fibrosis development. We therefore explored the underlying mechanism of the protective effect of mDASCs against pulmonary fibrosis caused by bleomycin. Hydroxyproline is a precursor of collagen, a key amino acid of collagen synthesis in the fibrotic lesions of mice. Compared to the control group, the injury group exhibited an obvious enhancement of hydroxyproline levels in lung tissues over time, consistent with previous studies [34, 35]. However, the treatment with mDASCs significantly decreased the hydroxyproline content in lung tissues. The development of bleomycin-induced pulmonary fibrosis was also related to the accumulation of α-SMA, which is a marker of myofibroblasts [36]. After bleomycin instillation, acute lung inflammation developed (day 7), and at this stage, we observed no change of α-SMA expression compared with the control group, but the expression of α-SMA increased on days 14 and 21, which were significantly alleviated by treatment with mDASCs.

In our study, bleomycin instillation markedly reduced O_2_ saturation and O_2_ partial pressure while it increased CO_2_ partial pressure in arterial blood on days 7, 14, and 21. However, the administration of mDASCs helped mice recover to a healthier post-injury pulmonary function with higher O_2_ saturation and O_2_ partial pressure, yet lower CO_2_ partial pressure. Consistent with this apparent protective effect in pulmonary function, the mDASCs improved the survival rates of mice injured by bleomycin.

We cannot exclude that the effects of mDASCs were indirect via induction of other molecules in the progression of fibrosis. For instance, the mDASCs exerted immunomodulatory effects that might influence CD45+ inflammatory cell infiltration in lung [14, 15]. Therefore, the mDASCs may play multiple roles (regeneration and/or immunomodulatory) in the progression of fibrosis and the specific mechanism remains an area of intense study.

## Conclusions

In summary, the mDASCs could ameliorate fibrosis, improve lung function, and reduce mortality of mice caused by bleomycin. This lung-protective effect is due to its process of lung regeneration and prevention of pulmonary fibrosis development. Therefore, DASCs may be a promising treatment for lung fibrosis.

## Additional files

Additional file 1: **Figure S1.** a Anti-KRT5 and anti-P63 immunofluorescence staining of lung section 7 and 21 days after administration of PBS. b Negative control of immunofluorescence staining. The lung section was stained by secondary antibodies without primary antibodies 7 and 21 days after administration of bleomycin. Scale bar, 20 μm. c Anti-KRT5 and anti-KI67 immunofluorescence staining of lung section 7 and 21 days after administration of PBS. Scale bar, 20 μm. (TIF 16715 kb)

Additional file 2: **Figure S2.** a Anti-GFP and anti-KRT5 immunofluorescence staining of lung section 21 days after bleomycin exposure treated with or without mDASCs. Scale bar, 100 μm. b Bright-field and direct fluorescence image of lungs from normal mice 7 days after transplantation of 1 × 10^6^ GFP-labeled mDASCs. c The weight changes of normal mice after transplantation of mDASCs. *n* = 3. Error bars, S.E.M. *ns*, not significantly different. d The lung wet-to-dry weight ratios of normal mice after transplantation of mDASCs. *n* = 3. Error bars, S.E.M. *ns*, no significantly different. e Lung sections from normal mice were stained by hematoxylin and eosin (H&E) 7 days after transplantation of 1 × 10^6^ GFP-labeled mDASCs. Scale bar, 200 μm. f Bright-field and direct fluorescence image of lungs 6 h (left) and 7 days (right) after transplantation of 1 × 10^6^ GFP-labeled mouse cervix basal cells or mDASCs. g The hydroxyproline content in the lung when mice were administrated by conditioned medium (CM) from mDASCs or control medium (with no cell) for 14 days after bleomycin injury. *n* = 3. Error bars, S.E.M. *ns*, no significantly different. (TIF 13826 kb)

## Abbreviations

DASCs: Distal airway stem/progenitor cells
IPF: Idiopathic pulmonary fibrosis
KRT5: Keratin-5
pCO_2_: CO_2_ partial pressure
pO_2_: O_2_ partial pressure
sO_2_: O_2_ saturation
W/D: Wet-to-dry weight
