# Supplementary figures and images for: Distal airway stem cells ameliorate bleomycin-induced pulmonary fibrosis in mice
Source: Stem Cell Res Ther. 2019 Jun 3;10:161. doi: 10.1186/s13287-019-1257-2 (PMC6547529; doi:10.1186/s13287-019-1257-2)

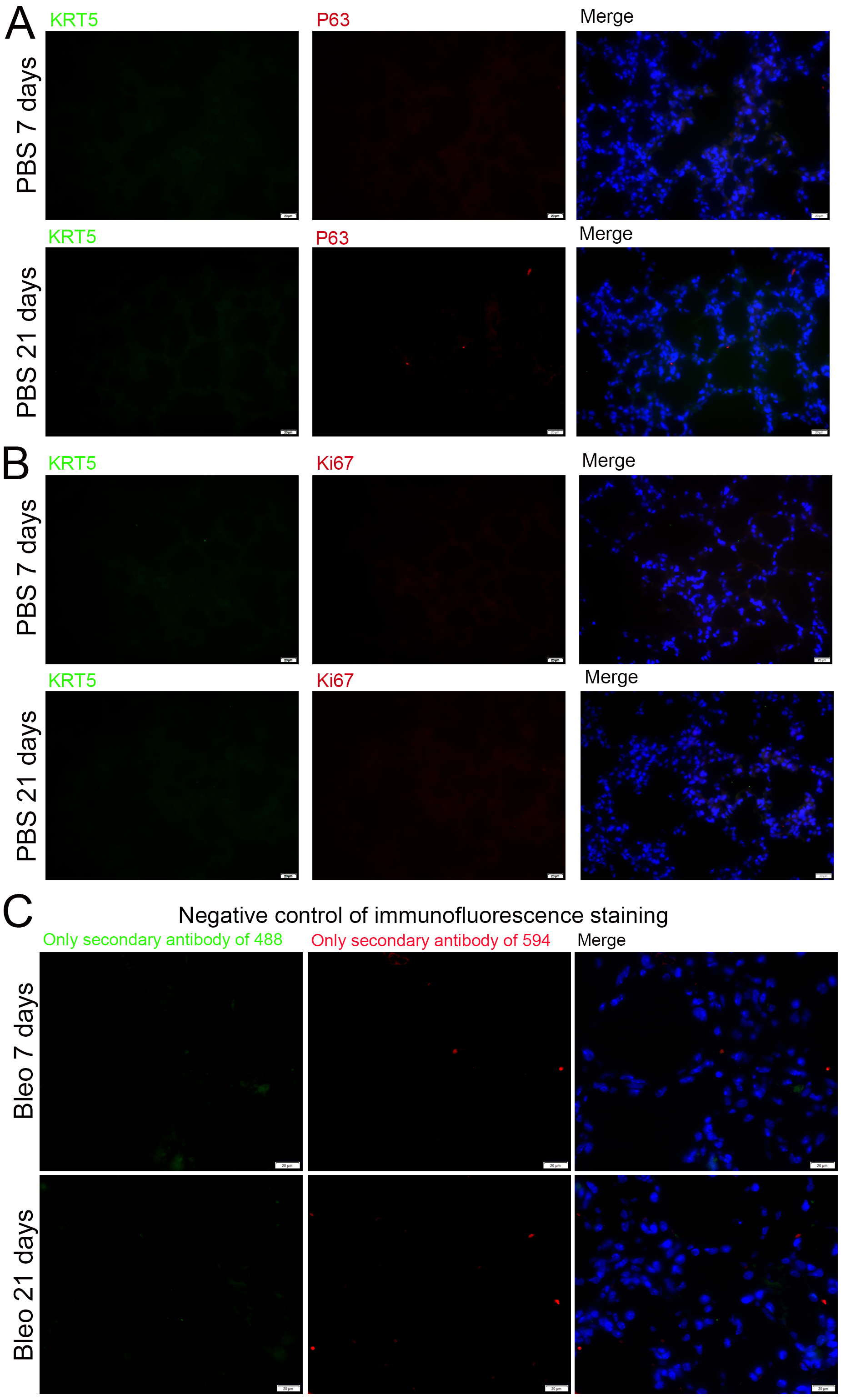

Supplement: Supplementary file 1 — Figure S1. a Anti-KRT5 and anti-P63 immunofluorescence staining of lung section 7 and 21 days after administration of PBS. b Negative control of immunofluorescence staining. The lung section was stained by secondary antibodies without primary antibodies 7 and 21 days after administration of bleomycin. Scale bar, 20 μm. c Anti-KRT5 and anti-KI67 immunofluorescence staining of lung section 7 and 21 days after administration of PBS. Scale bar, 20 μm. (TIF 16715 kb) [file 13287_2019_1257_MOESM1_ESM.tif]

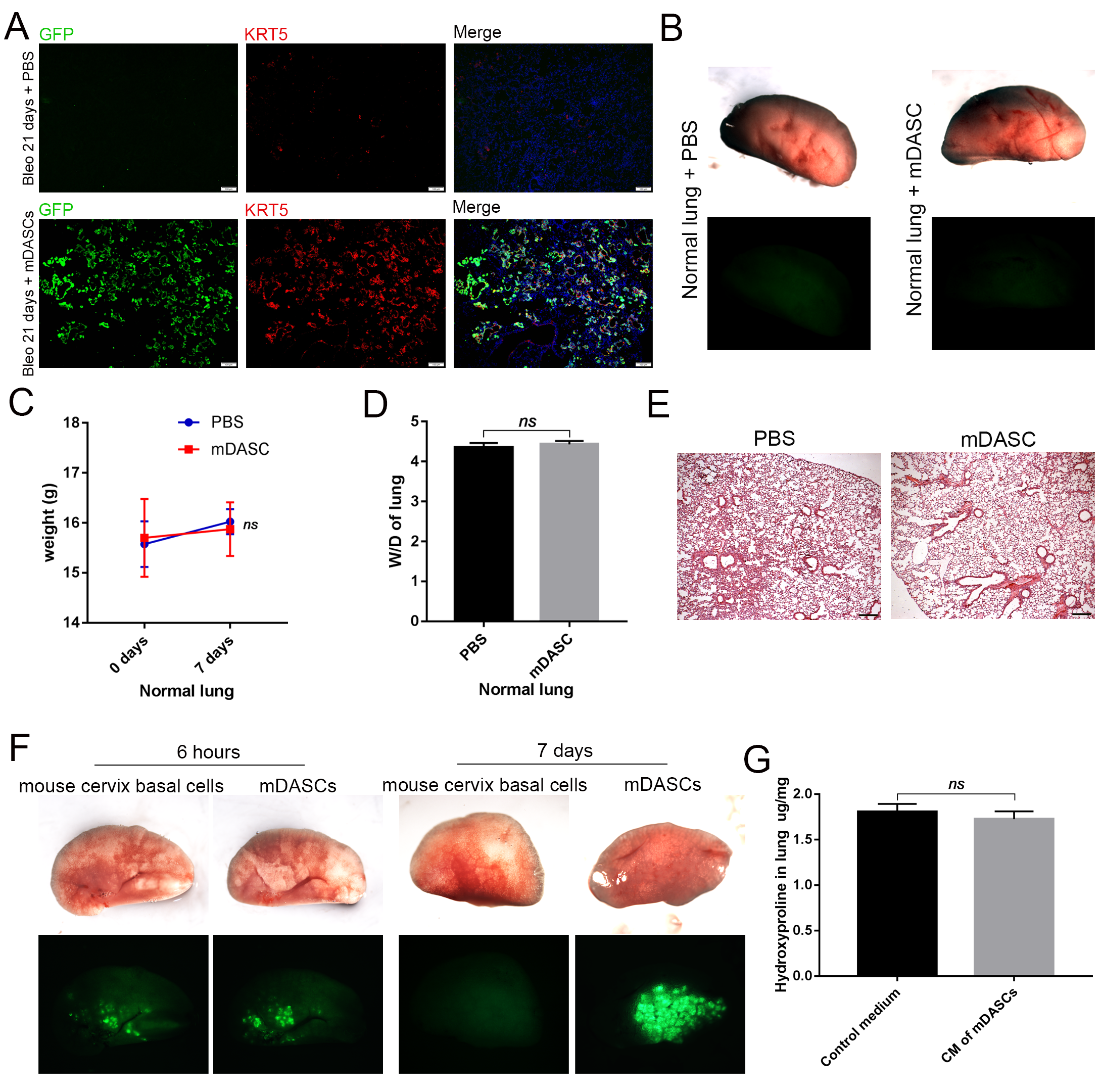

Supplement: Supplementary file 2 — Figure S2. a Anti-GFP and anti-KRT5 immunofluorescence staining of lung section 21 days after bleomycin exposure treated with or without mDASCs. Scale bar, 100 μm. b Bright-field and direct fluorescence image of lungs from normal mice 7 days after transplantation of 1 × 10^6^ GFP-labeled mDASCs. c The weight changes of normal mice after transplantation of mDASCs. n = 3. Error bars, S.E.M. ns, not significantly different. d The lung wet-to-dry weight ratios of normal mice after transplantation of mDASCs. n = 3. Error bars, S.E.M. ns, no significantly different. e Lung sections from normal mice were stained by hematoxylin and eosin (H&E) 7 days after transplantation of 1 × 10^6^ GFP-labeled mDASCs. Scale bar, 200 μm. f Bright-field and direct fluorescence image of lungs 6 h (left) and 7 days (right) after transplantation of 1 × 10^6^ GFP-labeled mouse cervix basal cells or mDASCs. g The hydroxyproline content in the lung when mice were administrated by conditioned medium (CM) from mDASCs or control medium (with no cell) for 14 days after bleomycin injury. n = 3. Error bars, S.E.M. ns, no significantly different. (TIF 13826 kb) [file 13287_2019_1257_MOESM2_ESM.tif]
